# Orofacial Pain: Molecular Mechanisms, Diagnosis, and Treatment 2021

**DOI:** 10.3390/ijms23094826

**Published:** 2022-04-27

**Authors:** Yoshizo Matsuka

**Affiliations:** Department of Stomatognathic Function and Occlusal Reconstruction, Graduate School of Biomedical Sciences, Tokushima University, 3-18-15 Kuramoto-cho, Tokushima 770-8504, Japan; matsuka@tokushima-u.ac.jp; Tel.: +81-88-633-7350

**Keywords:** orofacial pain, basic pain mechanisms, peripheral pain mechanisms, central pain mechanisms, management methodology, trigeminal system

## Abstract

The Special Issue “Orofacial Pain: Molecular Mechanisms, Diagnosis, and Treatment 2021” contains 6 articles published by 41 authors from different countries focusing on nucleus accumbens core GABAergic neurons, receptor-interacting serine/threonine-protein kinase 1, pannexin 1-mediated ATP signaling, ultra-low-frequency transcutaneous electrical nerve stimulation, and triamcinolone acetonide. The content covers several pain models, including neuropathic pain caused by peripheral nerve constriction or malpositioned dental implants, tongue cancer, myogenous temporomandibular dysfunction, and oral ulcerative mucositis. In addition, a review paper on trigeminal neuralgia is included.

Orofacial pain is one of the most severe pain problems worldwide, and many patients cannot find effective management methods. One review reported that the prevalence of orofacial pain is almost 25% of the population [1], and the etiology can be odontogenic or non-odontogenic. Non-odontogenic orofacial pain is challenging to diagnose. Orofacial pain is associated with important structures and functions, including aesthetics, speech, eating, and psychosocial status. In addition, orofacial pain varies from simple intraoral pain, such as toothache or periodontal disease, to more difficult management of diseases, such as myofascial orofacial pain, temporomandibular joint (TMJ) pain, orofacial pain attributed to lesions or disease of the cranial nerves, orofacial pain resembling presentations of primary headaches, idiopathic orofacial pain, and psychosocial assessment of patients with orofacial pain [2]. 

When exploring basic pain mechanisms, animal pain models should be developed because it is difficult to obtain samples from the human body. Animal pain models include acute pain, inflammatory pain, neuropathic pain, cancer pain, arthritic (joint) pain, muscle pain, postoperative pain, visceral pain, and other disease models [3,4]. Research on the basic mechanisms of orofacial pain is progressing. The management of orofacial pain is the most important factor in medicine and dentistry because orofacial pain is highly related to patient quality of life. It is difficult to manage pathological pain due to tissue injury or inflammation. Pain can occur in several locations, both in uninjured and injured areas. Pain sensation in normal uninjured areas may lead to misdiagnosis or inappropriate treatment. Thus, for appropriate pain management, we must first understand the underlying mechanisms of orofacial pain, including trigeminal nerve injury and orofacial region inflammation. 

This Special Issue, “Orofacial Pain: Molecular Mechanisms, Diagnosis, and Treatment 2021”, contains 6 articles published by 41 authors from different countries focused on basic orofacial pain mechanisms, diagnosis, and interventional modality. These articles utilized basic scientific research to establish the relationship between basic science and clinical work. This Special Issue features several animal pain models, including neuropathic pain caused by peripheral nerve constriction [5], malpositioned dental implants [6], tongue cancer [7], myogenous temporomandibular dysfunction [8], and oral ulcerative mucositis [9]. In addition, this Special Issue includes a review paper on trigeminal neuralgia [10].

Islam et al. targeted the nucleus accumbens core (NAcc) [5]. The NAcc is a neuronal region in the rostral basal forebrain that functions in the cognitive processing of rewards, pleasures, addictions, fears, etc. Islam et al. created a rat model of trigeminal neuropathic pain by infraorbital nerve constriction. This study reported that optogenetic stimulation of NAcc GABAergic neurons reduced neuronal firing in animals with neuropathic pain, and pain behavior significantly decreased. In addition, microdialysis showed that NAcc optical stimulation increased GABA, glutamate, acetylcholine, and dopamine while decreasing citrulline. Islam et al. concluded that NAcc stimulation can decrease trigeminal neuropathic pain [5].

Son et al. investigated the role of receptor-interacting serine/threonine-protein kinase 1 (RIPK1) in trigeminal neuropathic pain induced by inferior alveolar nerve injury with malpositioned dental implants in rats [6]. PIPK1 regulates cellular stress and inflammatory responses. After the induction of trigeminal neuropathic pain, the animals showed mechanical allodynia, and PIPK1 expression and TNF-α concentration were upregulated in the trigeminal nucleus caudalis. Intracisternal administration of Necrosatin-1 (RIPK1 inhibitor) reduced facial mechanical allodynia. Intracisternal injection of recombinant rat TNF-α in naive rats induced mechanical allodynia and upregulated RIPK1 expression in the trigeminal nucleus caudalis. In addition, there was co-localization of the TNF receptor and RIPK1 in astrocytes. Son et al. concluded that the TNF-α pathway activates RIPK1 in astrocytes and that RIPK1 pathway is a therapeutic target for orofacial neuropathic pain [6].

Koyama et al. studied the molecular mechanisms of tongue cancer pain and targeted pannexin 1 mediated ATP signaling in the trigeminal nucleus caudalis [7]. The authors reported that tongue cancer pain decreased after intracisternal administration of a pannexin 1 channel inhibitor. Pannexin 1 expression was upregulated in the trigeminal nucleus caudalis in the tongue cancer model. Some pannexin 1 immunofluorescence was colocalized with ionized calcium-binding adapter molecule 1 (Iba1) and glial fibrillary acidic protein (GFAP). Phosphorylation was observed in the trigeminal nucleus caudalis and an intracisternal pannexin 1 inhibitor inhibited phosphorylation. A P2X7 purinoceptor antagonist inhibited pain behavior and phosphorylation in the trigeminal nucleus caudalis. Koyama et al. concluded that tongue cancer causes pannexin 1 upregulation in the trigeminal nucleus caudalis microglia, and adenosine triphosphate released through pannexin 1 sensitizes nociceptive neurons [7].

Hsieh et al. explored the effects of ultra-low-frequency transcutaneous electrical nerve stimulation on alterations of biochemicals, electrophysiology, and jaw opening movement in a masticatory muscle pain model [8]. Electrical stimulation was repeatedly applied to the masticatory muscles. The maximum jaw opening increased, and endplate noise decreased after muscle stimulation. Muscle stimulation also significantly reduced substance P levels, increased μ-opioid receptors in the parabrachial nucleus, and increased c-Fos levels in the rostral ventromedial medulla. Hsieh et al. concluded that low-frequency muscle stimulation is a novel therapeutic approach for orofacial masticatory muscle pain [8].

Naniwa et al. studied the effect of triamcinolone acetonide on oral ulcerative mucositis pain in a rat model [9]. TRAFUL^®^ with triamcinolone acetonide decreased inflammatory responses with anti-inflammatory gene upregulation and decreased spontaneous pain behavior. TRAFUL^®^ combined with triamcinolone acetonide also decreased mechanical allodynia induced by oral ulcerative mucositis. Long-term pre-incubation with triamcinolone acetonide inhibited the hypertonic stimulation-induced Ca^2+^ response in dissociated trigeminal ganglion neurons. Naniwa et al. concluded that triamcinolone acetonide suppresses oral ulcerative mucositis pain through general anti-inflammatory actions and decreases mechanical pain sensitivity in the peripheral nerves [9].

Chen et al. reviewed the current evidence on trigeminal neuralgia pathophysiology and management [10]. They assessed the epidemiology, symptoms, classifications, pathophysiology, and management of TN. Chen et al. concluded that further studies are needed to focus on the basic mechanisms (genetics, molecular biology, electrophysiology, or imaging), and these studies will improve diagnosis and management of orofacial pain [10].
